# A scientific basis to determine the best paliative treatment for malignant pleural effusion

**DOI:** 10.1186/1749-8090-8-S1-O239

**Published:** 2013-09-11

**Authors:** A Hojski, A Crnjac

**Affiliations:** 1 Department of Thoracic Surgery, Division of Surgery, University Medical Centre Maribor, Maribor, Slovenia

## Background

The purpose of our study is to determine the role of growth factors (GF) in the process of pleurodesis. Biochemical characteristics of the pleural effusion (PE) are an important prognostic factor. Published data on the role of GF in the formation of PE and its treatment is somewhat contradictory.

## Methods

A prospective randomized study included 40 female patients with malignant PE resulting from breast carcinoma. They were divided into two groups and treated by means of mechanical (MP) or chemical (CP) pleurodesis. Samples of pleural fluid were analyzed by ELISA. The study was approved by the National Ethics Committee.

## Results

We found a 2.5 times increase in VEGF levels in the serum when performing a CP and a less than 25% increase of its original value in MP group. The average serum level of VEGF before receiving CP was 458.65ng/ml (172-702 ng/ml) and was elevated to a mean level of 1197.65ng/ml (406-2011ng/ml). In the MP group same results for serum VEGF were found 517.55 ng/ml (73-976 ng/ml) and 632.24 ng/ml (111 – 1136 ng/ml). Peak levels were noted between 24 and 48 hours after performing pleurodesis.

Of the GF secreted, particularly VEGF was present in excess. Serum VEGF could be the cause of some of the side effects we are faced with when performing CP.

The relationship between the released GF in MP is somewhat different. Namely, it has less influence on serum VEGF levels. We also believe that a proteoglycan bound FGF which is found in basal membrane might be the key to an effective pleurodesis.

## Conclusions

It is important to understand that the current knowledge of the effects of GF combined with our findings represents a contraindication for CP. Excess concentration of VEGF creates an environment that promotes the formation of PE, growth of tumor tissue and acts as a mediator of inflammation, possibly resulting in ARDS. We propose to use the MP as an effective, harmless and inexpensive method.

